# Physiological stress level and screening for malnutrition as preoperative predictors of postoperative complications in pancreatic surgery: a retrospective study

**DOI:** 10.1186/s12893-023-02062-y

**Published:** 2023-06-10

**Authors:** Igor A. Kryvoruchko, Plamen Staikov, Valeriy V. Boyko, Massimo Sartelli, Yulia V. Ivanova, Andrij Honcharov, Svetlana Gramatiuk, Karine Sargsyan

**Affiliations:** 1 Department of Surgery No.2, Kharkiv National Medical University, Nezalezhnosti Avenue, Kharkiv, 61022 Ukraine; 2 Krankenhaus Sachsenhausen, 60594 SchulstraßeFrankfurt Am Main, Germany; 3 Institute General and Emergency Surgery Named After V.T. Zaitcev of the National Academy of Medical Sciences of Ukraine, Balakireva Entry, Kharkiv, 61103 Ukraine; 4 Department of Surgery No.1, Kharkiv National Medical University, Balakireva Entry, Kharkiv, 61103 Ukraine; 5 Department of Surgery Macerata Hospital, Santa Lucia Street, 62100 Macerata, Italy; 6 Institute of Bio-Stem Cell Rehabilitation, Ukraine Association of Biobank, Puskinska Str, Kharkiv, 61022 Ukraine; 7 International Biobanking and Education, Medical University of Graz, Elisabethstraße, 8010 Graz, Austria

**Keywords:** Neutrophil–lymphocyte ratio, Nutritional risk index, Chronic pancreatitis, Cancer of the pancreas, Surgery, Outcomes

## Abstract

**Background:**

Assessment of ‘physiological stress levels’ and ‘nutritional status’ before surgery is important for predicting complications and indirect interventions on the pancreas. The aim of this study was to determine neutrophil–lymphocyte ratio (NLR) and nutritional risk index (NRI) indicators before surgery to predict 90-day complications and mortality in a cohort of patients with complicated chronic pancreatitis and cancer of the head of the pancreas.

**Methods:**

We evaluated preoperative levels of NLR and NRI among 225 subjects treated at different centres located in three countries. Short-term outcomes included length of hospital stay, postoperative complications, and mortality at 90 days and were appreciated based on NLR and NRI. The level of physiological stress was divided according by the formulas: neutrophil–lymphocyte ratio (NLR) = (neutrophil count, %)/(lymphocyte count, %). The nutritional state of the patients was divided according to the INR: NRI = (1.519 × serum albumin, g/L) + (41.7 × present weight, kg / usual weight, kg)].

**Results:**

All patients were operated. An analysis of the operations performed in three institutions demonstrated mortality in chronic pancreatitis and pancreatic pseudocysts in 1.4%, in chronic pancreatitis and the presence of an inflammatory mass mainly in the pancreatic head in 1.2%, and in cancer of the pancreatic head in 5.9%. The mean preoperative NLR was normal in 33.8% of the patients, the mild physiologic stress level was 54.7%, and the moderate was 11.5% before surgery. 10.2% of patients had a normal nutritional status, 20% had mild, 19.6% had moderate, and 50.2% had severe malnutrition. In a univariate analysis, at the cutoff of NLR ≥ 9.5 (AUC = 0.803) and the cutoff of NRI ≤ 98.5 (AUC = 0.801), increasing the risk of complications was observed (hazard ratio, 2.01; 95% CI, 1.247–3.250, *p* = 0.006), but at the cutoff of NRI ≤ 83.55 (AUC = 0.81), we observed a survival difference in operated patients (hazard ratio, 2.15; 95% CI, 1.334–3.477, *p* = 0.0025).

**Conclusions:**

Our study demonstrated that NLR and NRI were predictors of postoperative complications, but only NRI was a predictor of 90-day mortality in patients after surgery.

## Introduction

According to statistics, the number of patients with chronic, and pancreatic cancer has increased dramatically in recent years, both in Ukraine and abroad, as has the number of operations performed on these patients. Surgical treatment of these diseases is the most difficult problem in abdominal surgery, which is confirmed by the presence of a high number of postoperative complications, low resectability in cancer, high operational risk, unsatisfactory long-term treatment results, poor patient quality of life, etc. Modern ideas about the mechanisms of development of pancreatitis and tumour growth, despite studying the mechanisms of intracellular and extracellular signalling, do not allow us to unequivocally state the cause-and-effect relationships between the structural, functional, and clinical manifestations of inflammation on the one hand and the development of diseases on the other hand. The lack of convincing data on the relationship between inflammation and proliferative processes in the epithelium and stroma of the pancreas explains the existing debate on the issues of differentiation, diagnosis, prognosis complications, and the choice of surgical tactics in different diseases of the pancreas.

The quantitative indicators of blood leucocytes and the formula of leucocytes are used in clinical practice and are essential for diagnosing acute inflammatory diseases of various locations and etiologies. Currently, several indicators have been suggested for assessing the severity of inflammatory changes in the body and the effectiveness of therapy. One such indicator is the relationship between neutrophils and lymphocytes (NLR) as a marker of systemic inflammation and physiological stress levels. An analysis of recent literature has shown the predictive value of NLR in patients with oncological, cardiovascular, autoimmune, and infectious diseases, including chronic obstructive pulmonary disease, Alzheimer's disease, multiple sclerosis, schizophrenia [1–8], and COVID-19 [9]. Interesting is the study of this indicator in gastroenterology, in particular, its prognostic and diagnostic value in different patients [9–17]. Several previous studies have assessed the predictive role of inflammatory indicators in different diseases [18–21].

It was well known that malnutrition is a significant risk factor for morbidity and mortality after gastrointestinal surgery. The nutritional risk index (NRI) and the NLR are two well-tested tools that are used for diagnosis and prognosis outcomes in surgery [22–26]. For many years, the nutritional status of the patient before surgery was considered one of the key factors influencing the occurrence of various complications after surgery, and there are several studies on the possibility of using the NRI as a screening tool for malnutrition to predict postoperative complications and the impact on patient survival [27]. Currently, there is no predictive nomogram system for predictive screening among patients with pancreatic disease who are scheduled for surgery, both in patients with complicated chronic pancreatitis and in patients with pancreatic cancer [28]. On the one hand, most prognostic models are based on factors obtained after surgery; on the other hand, in several studies, the authors tried to use various non-tumor and tumor biomarkers associated with hepatopancreatobiliary diseases to assess the development of postoperative complications [28–31]. It is known that such non-tumour-biomarkers as the NLR and NRI are associated with the patient's preoperative inflammatory response to a pathological process in the pancreas, and, therefore, can facilitate the assessment of prognosis before surgery [32, 33]. It is also known that the disease of the pancreas is associated with severe nutritional problems with the development of malnutrition and the implementation of systematic nutritional support for these patients [34, 35]. Preoperative assessment of the diagnostic and prognostic value of NLR and NRI for predicting surgical risk may facilitate the identification of strategies to prevent postoperative complications and improve overall survival in various patient categories [36, 37].

**This study aimed** to determine how the preoperative NLR and NRI levels predict postoperative complications and mortality in direct pancreatic interventions for complicated chronic pancreatitis and pancreatic head cancer.

## Materials and methods

### Patient selection and data collection

A three-center retrospective study was conducted on 225 patients who were operated on with follow-up between January 1, 2014, and December 1, 2022, retrospectively evaluated in each of the 3 participating institutions in Ukraine (Kharkiv Regional Clinical Hospital and Institute Emergency and General Surgery named after V.T. Zaitcev) and Germany (Krankenhaus Sachsenhausen Hospital, Frankfurt am Main, Germany). The respective institutional review boards of each participating institution have approved this study. Demographic and clinical data were collected, including age, gender, BMI, diabetes mellitus, white blood cell count, total neutrophil and lymphocyte count, serum albumin, preoperative bilirubin level, preoperative biliary drainage, and preoperative nutritional support. Short-term outcomes included length of hospital stay, postoperative complications, and mortality at 90 days and were appreciated based on NLR and NRI.

This two-institution retrospective cohort study was handled in accordance with the Declaration of Helsinki. This manuscript adheres to the applicable STROBE guideline. The use of registered data follows the General Data Protection Regulation of the European Union.

The patient’s written Informed consent was signed for each bio-object from the residual materials.

The study and the use of data were consented to by the Ethics Committee of Kharkiv National Medical University, Ukraine (Protocol No. 6, November 11, 2022).

The level of physiological stress was divided according to the formulas: neutrophil–lymphocyte ratio (NLR) = (neutrophil count, percentages)/(lymphocyte count, percentages) [38, 39].

The nutritional state of the patients was divided according to the INR [40, 41]: NRI = (1.519 × serum albumin, g/L) + (41.7 × present weight, kg / usual weight*, kg)] *Usual weight is defined as stable body weight for last 6 months.

For both univariate analysis and the predictive model, cutoffs for NLR and NRI were calculated.

### Statistical analysis

The analysis was completed via IBM SPSS Statistics (https://www.ibm.com/products/spss-statistics).

We compared the reference characteristics of the patients with all the data. Missing data for serum albumin, current body weight, and usual body weight were excluded from the study to ensure the validity of the data. Differences in baseline characteristics using χ2 test for categorical variables for normally distributed or not normally distributed variables, respectively. Summary statistics were presented as integers and percentages for categorical variables and medians with interquartile ranges (IQRs) for continuous variables. The primary endpoint of interest was the presence of postoperative complications; the secondary endpoint was mortality within 90 days postoperatively, defined as the time interval between the date of surgery and the date of death or last follow-up, as appropriate. A *p*-value ≤ 0.05 was considered statistically significant. To determine the appropriate cutoff values, we used receiver operating characteristic (ROC) curves and determined the area under the curve (AUC). The efficacy of the model was considered limited at AUC ≥ 0.70; good at AUC ≥ 0.80; excellent at AUC ≥ 0.90. The predictive value of NLR and NRI for patients is carried out through univariate logistic regression analysis as predictive variables which were presented as risk ratios (HR) at 95% confidence intervals (CI). Overall survival within 90 was compared for various categories of interest using the Kaplan–Meier method with the log-rank test.

## Results

### Study characteristics and patient demographics

A total of 225 patients were retrospectively assessed and their demographic characteristics are presented in Table 1.

**Table 1:** Demographic, clinical, and laboratory characteristics of 225 patients

Indicators	Pathology of the pancreas			χ2/*P* value
	Chronic pancreatitis with pseudocyst of the pancreas (*n* = 71)	Chronic pancreatitis with inflammatory mass in the head of the pancreas (*n* = 86)	Cancer of the head of the pancreas (*n* = 68)	
**Age (years), median (IQR)**	55 [41–71]	56 [44–78]	59 [49–73]	1.376/0.503
**Sex**				12.469/0.014
**Male (%)/**	60 (84.5%)/11	78 (90.7%)/8	47 (69.1%)/21	
**female (%)**	(15.5%)	(9.3%)	(30.9%)	
**BMI кg/м**^**2**^**, median (IQR)**	26 [21–32]	22 [21–29]	21 [16–28]	1.179/0.555
**Diabetes mellitus**	12 (6.9%)	38 (44.2)	15 (22.1%)	8.752/0.013
**White blood cell count, × 10**^**3**^**, (%)**	7 [5.5–14.5]	6 [4–14]	6 [3.3–12]	0.369/0.832
**Total neutrophil count (%)**	58 [47–93]	67 [48–91]	66 [47–88]	0.927/0.629
**Total lymphocyte count (%)**	28 [9–37]	30 [8–36]	15 [5–33]	2.736/0.255
**Preoperative serum albumin (g/L)**	25 [22.2–33.6]	24 [18.8–37.5]	23 [21.4–33.8]	0.576/0.750
**Preoperative** **C-reactive protein** **(mg/l)**	45 [31.2–90.8]	38 [30.4–68.2]	54 [32.1–99.8]	4.913∕0.086
**Preoperative bilirubin (mmol/L)**	16 [2.3–33.4]	14 [5.2–56.8]	172 [18.9–455.7]	130.817/0.000
**Preoperative biliary drainage**	−	−	41 (60.3%)	NA
**Preoperative cysts drainage**	12 (16.9%)	−	−	NA
**Preoperative nutritional support**	6 (8.5%)	18 (20.9%)	31 (45.6%)	15.752/0.000

Abbreviations: *NA* Not applicable

In the group of patients with pancreatic head cancer, the main morphological type was volume-forming (*n* = 61, 89.1%). Characteristics of the features of the operations performed on the analyzed patients are presented in Table 2. Postoperative complications occurred in 12 patients (16.9%) with pseudocysts of the pancreas, in 13 (15.1%) with chronic pancreatitis, and in 37 (54.4%) with cancer. 6 patients (2.7%) died within 90 days after the operation. The median length of hospital stay was 10, 12, and 24 days, respectively (χ2 = 8.218, *p* = 0.017).

**Table 2:** Intra- and postoperative outcomes

Indicators	Pathology of the pancreas			χ2/*P* value
	Chronic pancreatitis with pseudocyst of the pancreas (*n* = 71)	Chronic pancreatitis with inflammatory mass in the head of the pancreas (*n* = 86)	Cancer of the head of the pancreas (*n* = 68)	
**Surgical result:**				
**An approach:**				65.173/0.000
open (%)	65 (91.5%)	86 (100%)	36 (52.9%)	
laparoscopic (%)	6 (8.5%)	0	32 (47.1%)	
**Type of surgery (%):**				NA
cysto-jejunostomy	55 (77.5%)	−	−	
cysto-gastrostomy	4 (5.6%)	−	−	
DPPHR according to Frey	12 (16.9%)	52 (60.5%)	−	
DPPHR according to Beger the Berne modification	−	26 (30.2%)	−	
Whipple’s procedure	−	8 (9.3%)	68 (100%)	
**Intraoperative data:**				
Duration of operation (min)	112 [55–143]	166 [143–245]	294 [234–850]	49.982/0.000
Red blood cell transfusion	0	4 (4.7%)	16 (23.5%)	
Portal venous resection	0	0	8 (11.8%)	
**Postoperative complications:**				
Clavien-Dindo I, n (%)	9 (25.3%)	8 (9.3%)	9 (13.2%)	34.321/0.000
Clavien-Dindo II, n (%)	2 (2.8%)	4 (14%)	18 (26.5%)	
Clavien-Dindo IIIa, n (%)	0	0	11 (16.2%)	
Clavien-Dindo IIIb, n (%)	0	0	2 (2.9%)	
Clavien-Dindo IVa, n (%)	0	0	1 (1.5%)	
Clavien-Dindo IVb, n (%)	0	0	1 (1.5%)	
Clavien-Dindo V, n (%)	1 (1.4%)	1 (1.2%)	4 (5.9%)	
**Delayed gastric emptying (ISGPF grade)**				
Grade A	1 (1.4%)	2 (2.3%)	3 (4.4%)	14.887/0.005
Grade B	0	1 (1.2%)	6 (8.8%)	
Grade C	0	4 (4.7%)	5 (7.4%)	
**Bile leakage (ISGLS grade)**				
Grade A	0	1	4 (5.9%)	13.805/0.008
Grade B	0	0	3 (4.4%)	
Grade C	0	2	1 (1.5%)	
**Pancreatic fistula (ISGPF grade)**				
Grade A	0	0	4 (5.9%)	34.617/0.000
Grade B	0	0	11 (16.2%)	
Grade C	0	4 (4.7%)	6 (8.8%)	
**Hospital stay** (day)	10 [8–15]	12 [11–34]	24 [19–154]	8.218/0.017

Abbreviations: *NA* Not applicable, *DPPHR* Duodenum-preserving pancreatic head resection, *ISGLS* International Study Group of Liver Surgery [42], *ISGPF* International Study Group of Pancreatic Fistula [43]

**Fig. 1:**
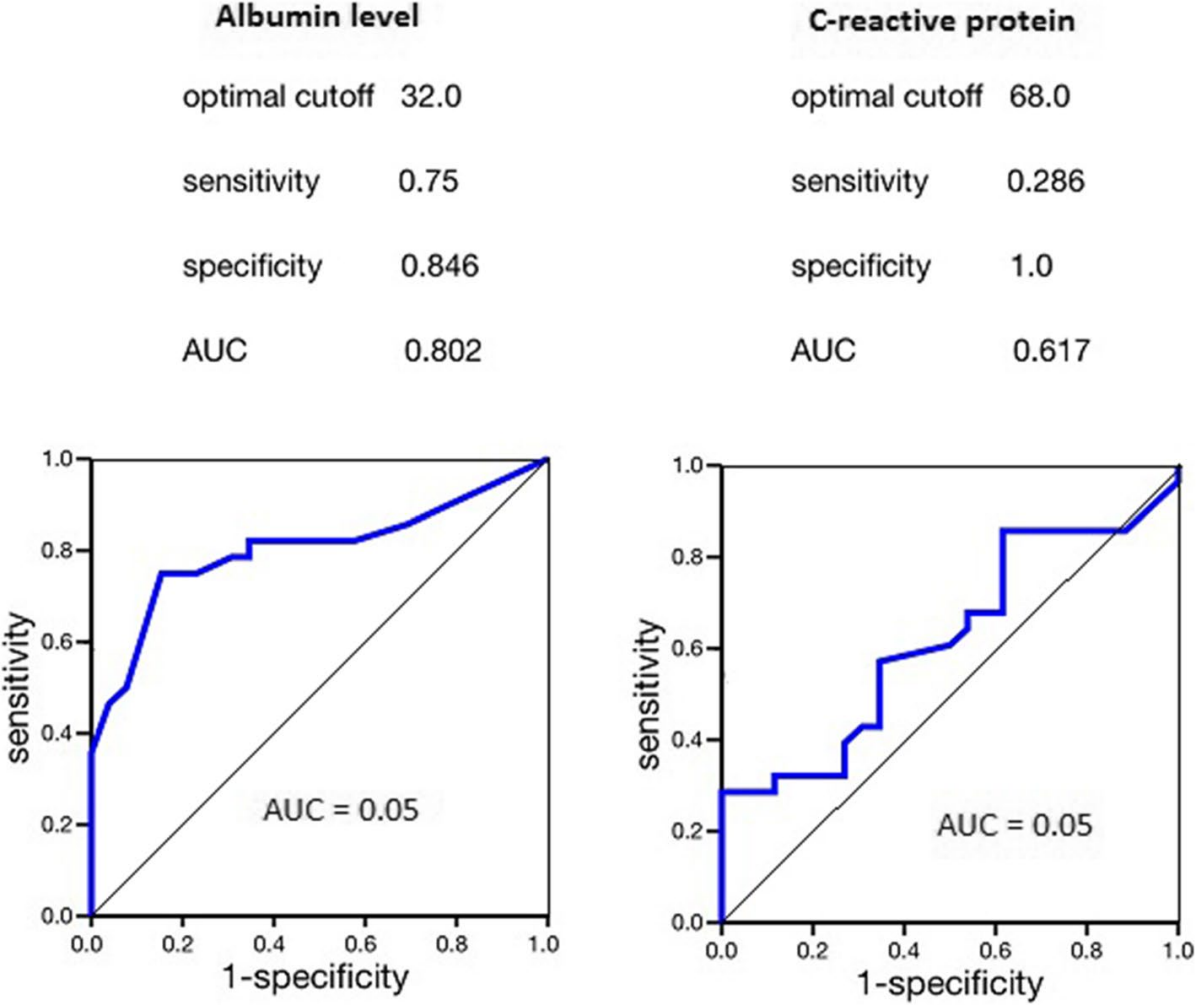
The area under the receiver operating characteristics curve for albumin level and C-reactive protein level in patients with complications after surgery

**Fig. 2:**
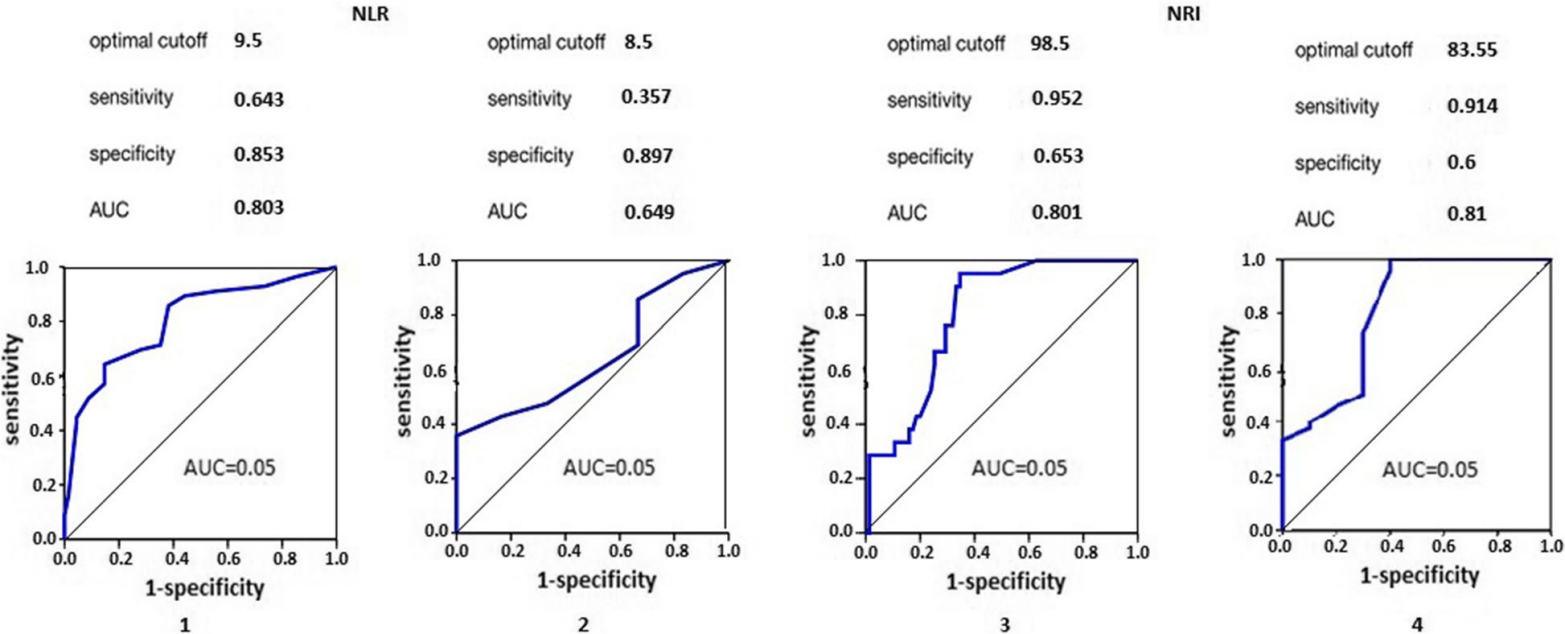
The area under the receiver operating characteristics curve for Neutrophil-Lymphocyte Ratio in patients with complications (1) and those who died (2), as well as the Nutritional Risk Index in patients with complications (3) and those who died (4 ) after surgery

The cutoff value for blood albumin was 32 g/l, and the preoperative area under the curve was better than that for C-reactive protein (area under the curve: 0.802 vs. 0.617, Fig. 1). However, there was no significant difference according to the χ2 criterion both in the average values of blood serum albumin (χ2 = 0.576∕*p* = 0.750) and in the level of C-reactive protein (χ2 = 4.913∕*p* = 0.086) in all types of pancreatic pathology before surgery. In addition, patients with albumin levels ≥ 32 g/l and C-reactive protein (CRP) ≤ 68 mg/l were more likely to have short-term complications (Clavien-Dindo I and II) after surgery (48% versus 52% and 42% versus 58%, χ2 = 0.162∕ *p* = 0.688) than patients with albumin < 32 g/l and CRP > 68 mg/l (46% versus 54% and 36% versus 64%, respectively, χ2 = 1.674∕*p* = 0.619). The mean preoperative NLR was normal in 33.8% of the patients, with mild physiological stress in 54.7% and moderate in 11.5% before surgery (Table 3). According to the NRI, 10.2% of patients had a normal nutritional status, 20% had mild, 19.6% had moderate, and 50.2% had severe malnutrition. Figure 2 depicts the ability of selected indicators NLR and NRI to predict complications and mortality in patients using ROC curve analysis. It should be noted that NLR had the optimal cutoff value of 9.5 points before surgery by criterion complications/non-complications (AUC 0.803, 95% CI 0.742–0.844, *p* = 0.0011), and by criterion survivors/non-survivors it had the optimal cutoff value of 8.5 points (AUC 0.649, 95% CI 0.509–0.745, *p* = 0.045); NRI had the optimal cutoff value of 98.5 points (AUC 0.801, 95% CI 0.738–0.841, *p* = 0.0013) by criterion complications/non-complications, and by criterion survivors/non-survivors its had the optimal cutoff value of 83.55 points (AUC 0.810, 95% CI 0.732–0.873, *p* = 0.0008) (Fig. 2). Using the predetermined cutoffs for survivors and non-survivors, 24.8% and 9.3% it was noted that overall survival was significantly shorter among patients with an NLR ≥ 8.5 than patients with an NLR < 8.5 in univariate (HR, 1.63 [95% CI, 1.01–2.647]; *p* = 0.056) and in NRI ≤ 83.55 than in patients with an NRI > 83.55 (HR, 2.31 [95% CI, 1.431–3.736]; *p* = 0.0009) analyses (Table 4). However, 11.6% and 60.8% of patients with complications after surgery with an NLR ≥ 9.5 than patients and in NRI ≤ 98.8 (HR, 2.01 [95% CI, 1.247–3.250]; *p* = 0.006) unlike the patients without complications (HR, 1.8 [95% CI, 1.112–2.905]; *p* = 0.022).

**Table 3:** Summary statistics of ratios

Ratio	Median	25%, percentile	75% percentile	Mean	SD	Cut point	The severity of the violations	Population (*n* = 225) with given cut point, no. (%)
**NLR**	2.0	1.0	5.0	2.35	1.36	≤ 5	Norm	76 (33.8)
	6.0	7.0	8.0	11.61	1.61	6–8	Mild	123 (54.7)
	12.0	10.0	15.0	6.67	0.7	9–18	Moderate	26 (11.5)
	−	−	−	−	−	> 18	Severe	−
**NRI**	102	100.7	106.4	102.5	2.3	> 100.0	Norm	32 (14.2)
	97.6	96.8	99.2	97.7	0.73	97.6–100.0	Mild	70 (31.1)
	88.7	83.8	95.4	89.41	3.99	83.5–97.5	Moderate	94 (41.8)
	72.8	66.4	83.1	74.59	5.02	< 83.5	Severe	29 (12.9)

Abbreviations: *NLR* Neutrophil to lymphocyte ratio, *NRI* Nutritional risk index

**Table 4:** Univariate proportional hazard models for overall survival (*n* = 225)

Indicators	HR	95% CI	*P*-value	χ2
NLR	1.63	[1.01, 2.647]	0.056	3.642
NRI	2.31	[1.431, 3.736]	0.0009	11.002
Without complications after surgery, *n* = 154	1.80	[1.112, 2.905]	0.0222	5.23
With complications after surgery, *n* = 71	2.01	[1.247, 3.250]	0.006	7.538
Survivors, *n* = 219	2.83	[1.738, 4.587]	0.00003	17.407
Non-survivors, *n* = 6	2.15	[1.334, 3.477]	0.00251	9.131

**Fig. 3:**
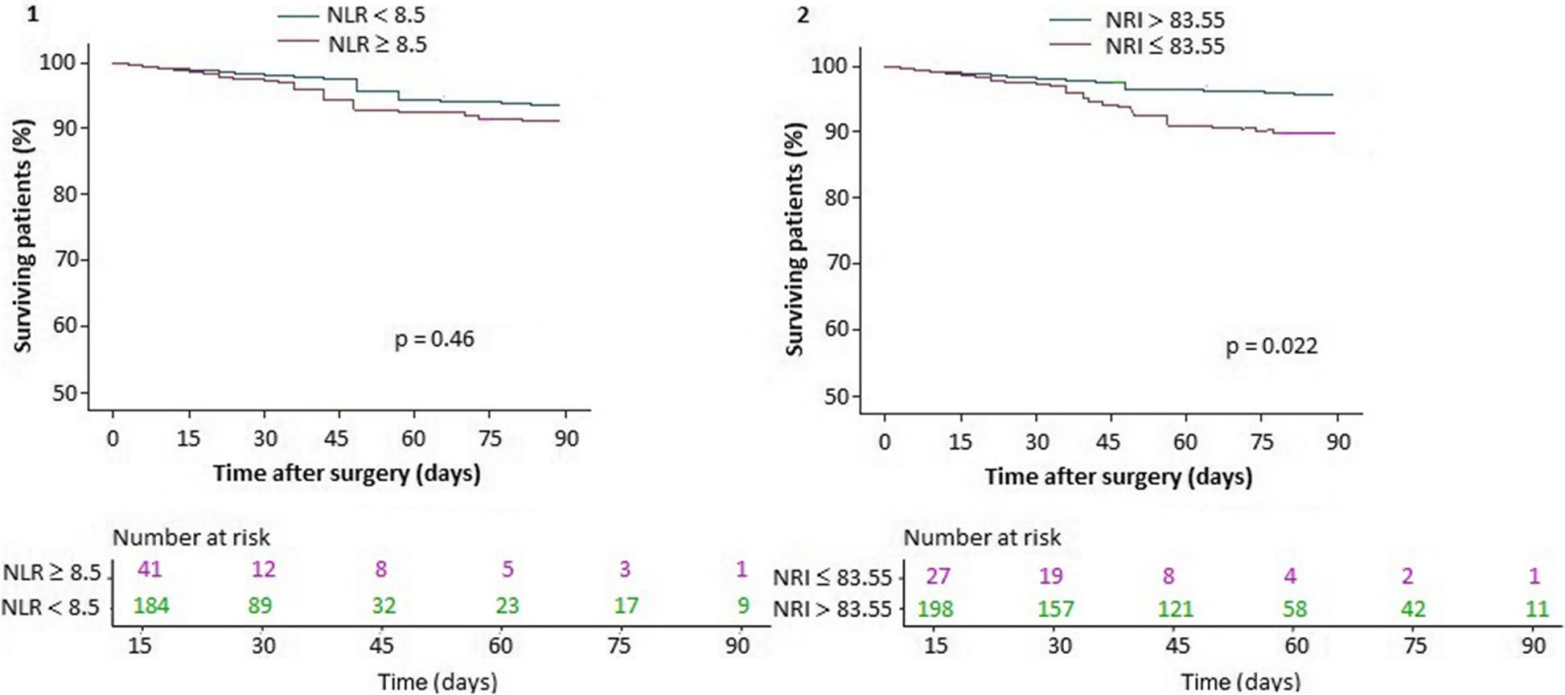
Cumulative Kaplan-Meier survival estimate over 90 days after surgery in Neutrophil-Lymphocyte Ratio (NLR, 1), and in Nutritional Risk Index (NRI, 2) in patients who died

Following the retro selection of survival predictors (Table 4), only the NRI remained an independent risk factor for the operational system in the model (HR: 2.31, 95% CI: 1.431–3.736; *p* = 0.0009). Other analyses have shown no “dose effect” of NLR on prognosis (HR: 1.63, 95% CI: 1.01–2.647; *p* = 0.056). Patients with a moderate and severe violation by data NRI died more often within 90 days compared with patients with a normal and mild violation (4.1% vs. 0.98%, *p* = 0.022). During the first fifteen days post-operatively the survival of patients with and without elevated NRI was equal (Fig. 3).

## Discussion

An analysis of the operations performed in three institutions demonstrated mortality in chronic pancreatitis and pancreatic pseudocysts in 1.4%, in chronic pancreatitis and the presence of an inflammatory mass mainly in the pancreatic head in 1.2%, and in cancer of the pancreatic head in 5.9%. That is, we have demonstrated that the use of various surgical interventions in this category of patients is not accompanied by high postoperative mortality, depending on the geographical location and volume of hospitals, but the number of development of postoperative complications is comparable to the figures given by other authors [44–47].

Our study examined the effect of preoperative NRI on 90-day mortality in a selected cohort of 225 patients, and this indicator, along with a high NLR, is also associated with the occurrence of postoperative complications in patients undergoing elective pancreatic surgery, as evidenced by studies conducted by other authors that different parameters in these patients were significantly associated with a higher rate of surgical complications and mortality [48–51].

When assessing the 90-day survival curve of NLR and NRI levels before surgery, it was determined that their values are statistically significant for predicting 90-day mortality after pancreatic surgery (*p* = 0.022). The optimal cutoff for NLR in the development of postoperative complications was determined before surgery and was 9.5 with a specificity of 85.3%, a sensitivity of 64.3% and an AUC of 0.803, which indicated a good sign of this selected indicator for the prognosis, but not satisfactory for the prognosis of mortality: the optimal cutoff was 8.5 with a specificity of 89.5%, a sensitivity of 35.7% and an AUC of 0.649. On the contrary, NRI had a good predictive value in the development of postoperative complications (optimum cutoff of 98.5 points with a specificity of 65.3%, a sensitivity of 95.2%, and an AUC of 0.801) and 90-day mortality with an optimal cutoff of 83.55 points, with a specificity of 60%, a sensitivity of 91.4%, and an AUC of 0.810. The combined use of the two indicators that were studied is possible as a screening tool to identify a group of patients with an increased risk of developing postoperative complications as well as 90-day mortality after surgery using NRI only.

In summary, the assessment of INR in patients undergoing pancreatic surgery for various pancreatic diseases was positive evaluated in this study to predict postoperative complications and mortality. It is helpful to determine NLI before surgery, although this indicator is not acceptable for predicting mortality up to 90-days after surgery based on our data.

Limitations of the research. This study has had several limitations. Firstly, it was a retrospective study, and our data was based on patient medical records that were processed. Secondly, not all patients were accounted for in this study, but only those with a full set of biomarkers in their study profile. Certainly, in the ignoring group have been patients who had died after surgery. As a result, bias in data selection could not be completely avoided and all results obtained require further verification in many more patients.

## Conclusions

Differences in local resources, opportunities for diagnostic and treatment procedures, institutional preferences, different experiences, and the severity of the disease all contribute to the variability in the effectiveness of a particular approach to the treatment of patients with complicated chronic pancreatitis and cancer of the pancreas. As shown in this study, the development of local and systemic complications of this disease, due to the individual characteristics of the patient's body, is of paramount importance for achieving clinically significant success after surgery. Preoperatively, low NRI as well as high NLR were significantly associated with higher rates of postoperative complications, and low NRI was a predictor of mortality in patients undergoing direct pancreatic interventions. Thus, the preoperative NRI and NLR values can be used to detect patients with possible postoperative complications, as demonstrated in the three centres where these patients were operated on.

## Data Availability

The datasets generated and/or analyzed during the current study are available from the corresponding author on reasonable request.

## Abbreviations

AUC: Area under curve
BMI: Body Mass Index
CI: 95% Confidence interval
DPPHR: Duodenum-preserving pancreatic head resection
HR: Hazard Ratio
IQR: Interquartile ranges
ISGLS: International Study Group of Liver Surgery
ISGPF: International Study Group of Pancreatic Fistula
NA: Not applicable
NLR: Neutrophil–lymphocyte ratio
NRI: Nutritional risk index
ROC: Receiver operating characteristic
